# Developing Interface Force Fields for Water and Oxygen on Pt, and *Pt*_*3*_ *Ni*, *Pt*_*3*_ *Co* Alloy Surfaces for Proton-Exchange Membrane Fuel Cell (PEMFC) Applications

**DOI:** 10.1021/acsomega.5c11427

**Published:** 2026-02-06

**Authors:** Aditya S. Kale, Gabriele Raabe

**Affiliations:** † Institut für Thermodynamik, 88774 Technische Universität Braunschweig, Braunschweig 38106, Germany; ‡ Cluster of Excellence SE2A-Sustainable and Energy-Efficient Aviation, TU Braunschweig, Braunschweig 38106, Germany

## Abstract

Interface force fields (IFFs) are crucial for the atomistic modeling of PEMFC interfaces. We benchmark ten optimization algorithms and four force-field forms against DFT adsorption energies and forces and present a framework for selecting the algorithm/force-field combination. At Pt–H_2_O, the best IFF yields a mean absolute error (MAE) of 0.32 eV for adsorption energy and 0.12 eV/Å^2^ for forces, cutting energy error by 70–90% and force error by 44–48% compared to COMB3, ReaxFF, and LJ mixing. Similar improvements are observed for Pt_3_Ni–H_2_O and Pt_3_Co–H_2_O. For Pt/Pt_3_Ni/Pt_3_Co–O_2_, energy MAEs are 0.30–0.68 eV and force MAEs are 0.42–0.85 eV/Å^2^; errors are larger for force components parallel to the surface, while normal components remain well captured, reflecting the challenges of describing O_2_ dissociation on (111) metal surfaces using classical force fields. The framework enables data-efficient IFF development and guides the selection of force-field forms and optimization algorithms for PEMFC and interfacial simulations.

## Graphical abstract

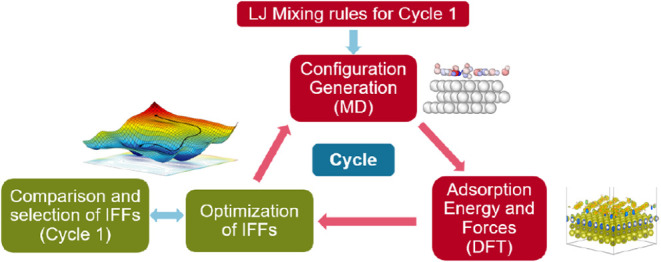

## Introduction

Understanding transport resistances at fluid–solid interfaces is crucial for controlling many critical steps in process intensification techniques, such as heterogeneous catalysis^1^ membrane separation, adsorption, and wetting, which are essential in the operation of batteries and fuel cells.^2^ A prominent example in which such interfacial phenomena are vital is the cathode catalyst layer (CCL) in proton-exchange membrane fuel cells (PEMFCs). There, voltage loss is caused by insufficient O_2_ transport, which is dominated by the configuration and reorganization of the ionomer at the catalyst–ionomer interface.^3^ The morphology of the ionomer, in turn, depends on many factors, such as ionomer composition, water content, temperature, etc. Although experimental techniques can probe certain aspects of these interfaces, molecular simulations allow one to gain mechanistic insight at the atomistic level. While the surface reaction chemistry at the catalyst surface itself is most reliably treated by quantum chemical or ab initio MD simulation, studies on the time and length scale required to capture the ionomer morphological organization and its effect on the O_2_ permeation resistance in the complex system consisting of the catalyst surface, the ionomer, water, hydronium, etc., are most effectively achieved through classical molecular dynamics (MD) simulations.^4^ Whereas most MD simulations of PEMFC cathode catalyst layers have focused on pure platinum or single-component surfaces,^5^ the use of alloy catalysts in MD studies remains largely unexplored due to the lack of suitable interface force fields (IFFs) for these more complex systems. Conventional force fields (FFs) often struggle to accurately model the interactions at solid surfaces, thus limiting their ability to reliably generate equilibrium atomic configurations—a prerequisite for accurate prediction of dynamic properties such as diffusion.^6^ Therefore, the development of IFFs is crucial to improving the accuracy and applicability of MD studies involving fluid–solid or heterogeneous interfaces.^7^

Previously, metal–fluid interface force fields (IFFs) were typically obtained by first parametrizing a force field for the metal phase and then coupling it to an existing bulk-fluid force field via mixing rules to reproduce interfacial properties.^8,9^ This approach is valid for some interfaces between metals with a face-centered cubic structure and organic compounds, but not for others.^10,11^ Ercolessi and Adams first presented the “force matching” approach to develop interatomic FFs,^12^ which involves fitting the forces acting on individual atoms in various reference structures, as well as cohesive energies and stresses on the unit cell obtained from *ab initio* calculations. Johnston et al. parametrized IFFs by matching interaction energies of adsorbed fluid molecules on the surface, as calculated by density functional theory (DFT),^14,15^ using a genetic algorithm.^13^ Valadez Huerta and Raabe proposed a parametrization of IFFs that considered interatomic forces as well.^16^ Their method also accounted for bulk effects, i.e., interactions between fluid molecules adsorbed on the surface. In such parametrization strategies, adsorption energy is often chosen over absolute total energy because it isolates the interaction between the adsorbate and the surface, removing bulk contributions from the slab and fluid that are irrelevant to interfacial modeling. This ensures that the optimized parameters reproduce the energetics of the interface rather than being dominated by large cohesive energy terms; such an approach is consistent with best practices in prior metal–adsorbate FF developments.^17^ The amount of computationally expensive training data that is usually needed for IFFs was also addressed and optimized for their method.

To develop IFFs,^18–22^ the process typically begins with the selection of an appropriate functional form for the FFs to model van der Waals (vdW) interactions. Common choices include the Buckingham potential,^23^ the Morse potential,^24^ the Born–Mayer–Huggins (BMH) potential,^25^ the Mie potential,^26^ and the Lennard-Jones (LJ) potential,^27^ among others. Computational optimization algorithms are then utilized to parametrize these FFs to reproduce interatomic interactions as determined by DFT calculations. Although this process may seem straightforward, finding the optimal combination of the force field functional form and optimization algorithm can be quite challenging. Sen et al. compared multistart local optimization algorithms, such as the Simplex, Levenberg–Marquardt, and POUNDERS methods, with single- and multiobjective genetic algorithms (GAs) to parametrize the Morse FF to reproduce structural, thermodynamic, and mechanical properties of IrO_2_ crystal polymorphs computed using DFT.^28^ Hence, the DFT training set included unit cell energies, lattice constants, internal coordinates, and elastic constants of various phases of IrO_2_. They found that employing 500 random initial guesses allowed the local optimization algorithms to obtain solutions up to 380,210 times better than those found by the genetic algorithms. Using a larger population for GAs required more generations (iterations) to achieve convergence. Interestingly, they found that the optimization methods delivered more accurate predictions for a test set generated with known FF parameters than for properties derived from DFT calculations. This discrepancy underscores the limited generalizability of these strategies and highlights the urgent need for a systematic comparison of both FFs and their optimization algorithms.

More recently, Gangan et al. compared conjugate gradient (CG), Broyden–Fletcher–Goldfarb–Shanno (BFGS), and Nelder–Mead (NM) methods to parametrize Stillinger–Weber (SW) and Environment-Dependent Interatomic Potentials, where only four initial guesses were used for the local optimization algorithms—a relatively small number that may limit exploration of the parameter space.^29^ Berg et al. optimized three different varieties of LJ, Buckingham, and Morse FFs using the BFGS algorithm.^18^ Similarly, Fracchia et al. optimized different FFs of varying complexity using the differential evolution algorithm.^19^ Both studies highlight the significant impact that the complexity of FFs and the selection of descriptors have on their accuracy; however, a comparative analysis of the performance of different optimization algorithms was not performed in these studies. This is necessary, even though more complex force fields can theoretically provide a better description of nonbonded interactions,^19^ they also introduce more parameters and increase the complexity of the objective function. As a result, certain optimization algorithms may struggle to find solutions efficiently when faced with a high-dimensional and intricate parameter space.^30,31^ This challenge has also been recognized by other researchers,^32,33^ yet it remains an underexplored problem in the field. Systematic benchmarking has been conducted to assess various machine learning (ML)-based FF models,^34^ where the quality of each ML model was assessed based on the mean absolute error in force prediction and stability. Therefore, it is crucial to perform such benchmarking and to evaluate the performance of both the different classical functional forms of force fields and optimization algorithms together in order to achieve the most accurate and reliable results.

In light of these considerations, the present study sets out to benchmark ten different optimization algorithms, both local and global, to parametrize four distinct functional forms for an IFF: BMH, Morse, Buckingham, and Mie. The procedure for generating the initial training data set and the sequential improvement of IFFs through data set enrichment is adapted from the work of Valadez Huerta and Raabe.^16^ We incorporate best practices from previous benchmarking research^35–37^ to ensure that each optimization routine is evaluated not only for efficiency but also for reliability. Our work addresses the shortcomings found in current approaches by systematically employing multistart local algorithms alongside global algorithms. To complement these classical methods, we also train a DeepMD model.^38^ To establish a workflow for generating consistent IFFs for all components of the CCL, the methodology is initially developed and tested here for adsorbed H_2_O and O_2_ on (111) surfaces of Pt, Pt_3_Ni, and Pt_3_Co. Water and oxygen are the most abundant fluid molecules in the CCL of PEMFCs, while platinum, nickel, and cobalt alloys with (111) surfaces are among the most widely used catalysts due to their high current density output.^5^ We compare the final IFFs to those available in the literature and further validate them against DFT calculations and large-scale simulations. We test the applicability of the optimized IFFs for predicting stable configurations of H_2_O and O_2_ in the adsorption region on pure Pt and alloyed surfaces, which are very important in order to study dynamic properties like diffusion.

## Methods

### Data Set Generation: Configurations, Adsorption Energies and Forces, and Force Fields

The benchmarking procedure is evaluated on Pt–H_2_O and Pt–O_2_ interfacial systems and then applied to the interface of H_2_O and O_2_ with Pt_3_Ni and Pt_3_Co alloy surfaces. As a representative example, we describe in detail the procedure for generating configurations of H_2_O adsorbed on the Pt(111) surface.

During all MD calculations, the interatomic forces between atoms of Pt, Pt_3_Ni, and Pt_3_Co slabs are evaluated using the second nearest-neighbor modified embedded atom method (MEAM) force field.^39^ The MEAM FF is given by eq 1, in which *F*_*i*_ is the embedding energy as a function of atomic electron density ρ, and ϕ is a pair potential interaction. The interaction is summed over all neighbors *j* of atom *i* within the cutoff distance, and *r*_*ij*_ is the distance between atoms *i* and *j*

1 $$u^{\mathrm{MEAM}}\left(r_{ij}\right)=\underset{i}{\sum }F_{i}\left({\mathrm{\rho ̅}}_{i}\right)+\frac{1}{2}\underset{i}{\sum }\underset{j\neq i}{\sum }\phi_{ij}\left(r_{ij}\right)$$

Coulombic interactions are calculated using eq 2, where *q*_*i*_ and *q*_*j*_ are charges on atoms *i* and *j*, and ϵ_0_ is the vacuum permittivity

2 $$u^{\mathrm{Coulomb}}=\frac{1}{4\pi \varepsilon_{0}}\frac{q_{i}q_{j}}{r_{ij}}$$

The F3C water force field by Levitt et al. is used to describe the H_2_O bulk^40^ and oxygen force field by Wang et al. is used to describe the O_2_ bulk.^41^ These FFs include intramolecular energy contributions for bonds (*b*) and angles (*a*) (only for O_2_)­

3 $$u_{b}\left(r\right)=k_{b}{\left(r-r_{0}\right)}^{2}$$

4 $$u_{a}\left(\theta \right)=k_{a}{\left(\theta -\theta_{0}\right)}^{2}$$

and the van der Waals Lennard-Jones (LJ) energy

5 $$u^{\mathrm{LJ}}\left(r_{ij}\right)=4\epsilon \left[{\left(\frac{\sigma }{r_{ij}}\right)}^{12}-{\left(\frac{\sigma }{r_{ij}}\right)}^{6}\right]$$

as well as the Coulombic contribution of eq 2. The parameters *k*_*b*_ and *k*_*a*_ are force constants, and *r*_0_ and θ_0_ are the respective equilibrium bond lengths and angles. Full parameter details for H_2_O and Pt bulk force fields are provided in Table  S1.

The parameterization procedure was tested on a Pt–H_2_O interface using a three-layer Pt(111) slab. The cubic lattice parameter was set^42^ to *a* = 3.924 Å, so that each in-plane vector along [1̅10] and [11̅0] has length $\frac{a}{\sqrt{2}}\approx$ 2.773 Å.

We chose a three-layer 4 × 4 surface supercell slab with the (111) plane normally aligned along the *x*-axis. Two identical, *ab initio*-optimized slabs were then placed in the simulation box in parallel, forming a “capacitor”-like arrangement with two vacuum gaps of 40 Å and 20 Å between the slabs (see Figure 1). The larger gap contains the fluid phase for the interface of interest, while the smaller gap on the opposite side is introduced solely to prevent direct interactions between periodic images of the slabs along the *x*-axis. In all DFT calculations, only the energies and forces for the target adsorption region (larger-gap interface) were considered. The clean interface in the smaller vacuum gap remained inactive and did not affect the training data due to the large separation, which removed residual interactions between periodic replicas.

**Figure 1:**
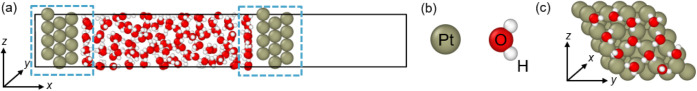
(a) Snapshot of the Pt–H_2_O capacitor system after MD. The blue dashed box indicates the region extracted for the *ab initio* calculations. (b) Denominations of different atom types used throughout the study. (c) Exemplary configuration of H_2_O adsorbed on the left slab, taken after 500,000 steps of MD. Images are generated using OVITO.^43^

Separately, a bulk H_2_O phase was equilibrated in the NVT ensemble by using classical MD. The simulation box dimensions were chosen to exactly span the larger vacuum gap in the dual-slab capacitor setup, with an additional 3.5 Å separation between the bulk and each of the surfaces. A total of 135 H_2_O molecules were included to reproduce the experimental density of liquid water at 298.15 K and 1 bar. The equilibrated water slab was then inserted into the larger interslab gap to complete the Pt–water interfacial model.

A classical MD run of the complete Pt–H_2_O capacitor system was carried out at 298.15 K and 1 bar. An example snapshot after equilibration is shown in Figure 1a, together with the labeling scheme for atom types (Figure 1b) used throughout this work. The shaded box in Figure 1a indicates the interfacial region that is extracted for the *ab initio* calculations. Keeping the overall box dimensions fixed, only the particles within this marked volume are retained, yielding multiple interfacial configurations containing 11–13 H_2_O molecules adsorbed on the Pt(111) surface. To ensure diverse sampling of H_2_O configurations, six snapshots—three from the “left” slab and three from the “right” slab—are extracted at timesteps 0, 500,000, and 1,000,000 from the MD trajectory. One of these is shown in Figure 1c.

To initialize the optimization procedure at the first “cycle” (see the next subsection for definition), the interatomic interactions between the Pt slab and the H_2_O bulk are described with an LJ force field (eq 5) and arithmetic mixing rules, with ϵ and σ values for Pt taken from the literature.^9^ During IFF optimization, four different FFs are evaluated: Born–Mayer–Huggins (BMH, eq 9), Morse (eq 7), Mie (eq 6), and Buckingham (eq 8)­

6 $$u^{\mathrm{Mie}}\left(r_{ij}\right)=\epsilon \left[\frac{n}{n-m}{\left(\frac{n}{m}\right)}^{m/n-m}\left({\left(\frac{r_{e}}{r_{ij}}\right)}^{n}-{\left(\frac{r_{e}}{r_{ij}}\right)}^{m}\right)\right]$$

7 $$u^{\mathrm{Morse}}\left(r_{ij}\right)=D_{0}\left[{\left(1-e^{-a(r_{ij}-r_{0})}\right)}^{2}-1\right]$$

8 $$u^{\mathrm{Buck}}\left(r_{ij}\right)=A\exp\left(-\frac{r_{ij}}{\rho }\right)-\frac{C}{r_{ij}^{6}}$$

9 $$u^{\mathrm{BMH}}\left(r_{ij}\right)=A\exp\left(\frac{\sigma -r_{ij}}{\rho }\right)-\frac{C}{r_{ij}^{6}}-\frac{D}{r_{ij}^{8}}$$

Coulombic interactions are considered with each FF according to (eq 2).

These FFs are selected to demonstrate a range of complexities and physical models for atomic and molecular interactions. The Mie FF generalizes the LJ FF with tunable repulsive (*n*) and attractive (*m*) exponents. The Morse FF provides an exponential form to capture the anharmonicity of atomic interactions, especially bond stretching and dissociation. The Buckingham FF describes nonbonded interactions as the sum of an exponential repulsive and an *r* ^–6^ attractive (London dispersion) term, which provides a more realistic treatment of electron cloud overlap than polynomial forms.^23^ The Born–Mayer–Huggins (BMH) potential extends this by including an *r* ^–8^ attractive term—representing dipole–quadrupole dispersion interactions—in addition to the exponential repulsion and sixth-power attractions. Parameter limits for all force fields are given in Table 1.

**Table 1:** Typical Lower and Upper Bounds for Parameters in Buckingham, BMH, Morse, and Mie FFs

Parameter	Lower	Upper	Units	Force Field
*A*	0	1 × 10^7^	kcal/mol	Buckingham, BMH
*C*	0	1 × 10^4^	kcal· Å^6^/mol	Buckingham, BMH
*D*	0	1 × 10^4^	kcal· Å^8^/mol	BMH
ρ	0.1	10.0	Å^–1^	Buckingham, BMH^a^
*D*_0_	0.1	1 × 10^3^	kcal/mol	Morse
α	0.5	10.0	Å^–1^	Morse^b^
ϵ	0.001	1 × 10^3^	kcal/mol	Mie
*r*_0_, σ	0.7^c^	2.36^d^	Å	BMH, Mie, Morse
*n*	8	20	—	*n* > *m* ^e^; Mie
*m*	4	8	—	Mie

a Limits for ρ adopted from original works.^23,25^

b Limits for α from ref. ^24^.

c Sum of atomic radii of the two atom types considered.

d Distance greater than or equal to the distance of the most stable configuration on the surface (if available); else, 5.0 Å.

e The repulsive exponent (*n*) must be greater than the attractive exponent (*m*).

Except for parameters, such as σ and *r*_0_, which have physical meaning as the distance at which the interaction energy between two atom types is zero, most force field parameters, particularly pre-exponential and exponential repulsive terms in empirical FFs, are just fitting constants. Their ranges are set broadly, guided by prior literature and stability considerations, to ensure a comprehensive parameter space search during empirical optimization.^44–47^

Configurations for the alloys Pt_3_Ni and Pt_3_Co, and for the other fluid O_2_, are generated using the same protocol. For alloy slabs, atomic distributions for Pt, Ni, and Co are chosen to reflect the experimental surface compositions reported by Stamenkovic et al. and Kobayashi et al. The first atomic layer is 100% Pt, the second about 50% Ni for Pt_3_Ni and 100%Co for Pt_3_Co, and from the third layer on, the bulk composition is 75% Pt and 25% Ni (Pt_3_Ni) or Co (Pt_3_Co).^48,49^ These Pt-skin slabs are modeled using the MEAM force field. LJ parameters for O_2_, Ni, and Co bulk FFs used for initial interface FFs are given in Table  S1 and are obtained from literature.^9,41,50^

### Computational Details

All density functional theory (DFT) calculations were performed with Quantum ESPRESSO (version 7.1).^51,52^ Electronic exchange–correlation effects were treated using the Perdew–Burke–Ernzerhof (PBE) generalized gradient approximation (GGA).^53^ Core–valence interactions were modeled with ultrasoft pseudopotentials (USPPs)^54^ from the Dal Corso PSLibrary (v1.0.0) distributed with Quantum ESPRESSO.

A plane-wave kinetic-energy cutoff of ecutwfc = 50 Ry (≈ 680 eV) and a charge–density cutoff of ecutrho = 600 Ry (≈ 8160 eV) were used, chosen from convergence tests ensuring total-energy changes <1 × 10^–4^ eV/atom and force changes <0.01 eV/Å. Electronic occupations were treated using Marzari–Vanderbilt smearing^55^ with degauss = 0.00735 Ry (0.10 eV). Dispersion interactions were included via the Grimme D3 scheme with zero damping.^56^ SCF iterations used a convergence threshold of conv_thr = 1.0 × 10^–4^ Ry and Thomas-Fermi preconditioned mixing with mixing_beta = 0.2.

#### vc-relax: Lattice Optimization

Lattice optimizations (vc-relax) were performed for Pt, Pt_3_Ni, and Pt_3_Co metal slabs comprising three atomic layers with four atoms per layer (total: 12 atoms), as illustrated in Figure 2. All slabs were terminated by a (111) surface: for Pt and Pt_3_Ni slabs, the surface normal was aligned along the *x*-axis; for Pt_3_Co slabs, the normal was aligned along the *z*-axis to probe directional effects.

**Figure 2:**
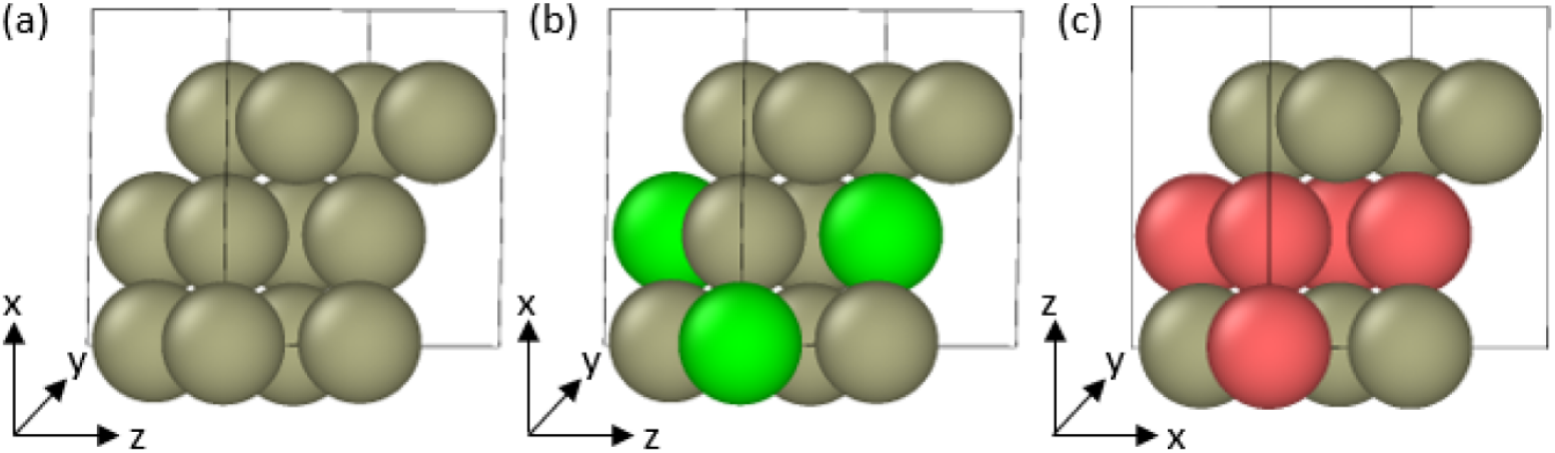
vc-relaxed (111)-terminated metal cells used as adsorbents: (a) Pt, (b) Pt_3_Ni, and (c) Pt_3_Co. Atomic representation: brown atoms are Platinum, green are Nickel, and red are Cobalt. The surface normal is aligned with *x* in (a,b) and with *z* in (c), as indicated by the axes, to probe directional effects. Unit cell outlines are shown. These optimized lattices serve as the unit cells of the adsorbent in subsequent adsorbate–adsorbent–vacuum configurations.

Both ionic and cell (lattice) degrees of freedom were relaxed using BFGS dynamics until residual Hellmann–Feynman forces were below 5 × 10^–4^ Ry/Bohr and total-energy changes were below 1 × 10^–5^ Ry. Brillouin-zone sampling employed Γ-centered Monkhorst–Pack meshes of 3 × 5× 4. The resulting relaxed lattice parameters are summarized in Table 2.

**Table 2:** vc-relaxed Lattice Parameters for Three-Layer (12-Atom) (111) Metal Cells^a^

**System**	***a*** (Å)	***b*** (Å)	***c*** (Å)	α (^◦^)	β (^◦^)	γ (^◦^)
Pt_3_Ni	6.71657	5.49769	5.49769	119.6530	89.9722	90.0277
Pt	6.88797	2.81200	2.81200	119.99	90.0	90.0
Pt_3_Co	5.41740	5.41740	6.45943	90.0	90.0	120.0

a Pt and Pt_3_Ni have the surface normal along *x*; Pt_3_Co along *z*.

#### Single-Point SCF

For adsorption-energy and force calculations, we considered slab-fluid configurations extracted from classical MD trajectories. Each selected MD snapshot yields two independent “configurations” in the present work; an example is shown in Figure 1c. From the dual-slab “capacitor” model of Figure 1a, only atoms within the region bounded by the blue dashed lines were retained. The full simulation cell dimensions were kept, thereby including vacuum spacing sufficient to avoid spurious interaction between periodic slab images. For each configuration, single-point SCF calculations were performed three times: first only for the adsorbate (slab), second only for the adsorbent (H_2_O/O_2_), and third for the adsorbate and adsorbent, using the same ecutwfc, ecutrho, pseudopotentials, smearing method, dispersion correction, and SCF convergence criteria as in the vc-relax runs. In contrast to the lattice-optimization calculations, the Brillouin-zone sampling employed Γ-centered Monkhorst–Pack meshes of 1 × 3 × 3 for Pt and Pt_3_Ni slabs, and 3 × 3 × 1 for Pt_3_Co slabs, reflecting the elongated vacuum direction for each system. No atomic positions or cell parameters were relaxed in these SCF runs; total energies and Hellmann–Feynman forces were extracted directly for use in adsorption-energy and force calculations. Input files for single-point SCF calculations in the first fitting cycle for the Pt_3_Ni–H_2_O system—including atomic coordinates, k-point meshes, pseudopotential filenames, and computational parameters—are provided in the Supporting Material.

All classical molecular dynamics (MD) simulations were performed using LAMMPS.^57^ The positions of all slab atoms (Pt, Pt_3_Ni, and Pt_3_Co) were held fixed throughout the MD runs. This constraint was imposed because the second-nearest-neighbor MEAM parametrization employed for the slabs is optimized for cohesive energies and static structures and does not reliably reproduce dynamical relaxations of metallic atoms in extended surfaces. Interactions were evaluated using the minimum-image convention with a real-space cutoff of 8 Å. Covalent bonds in the fluid phase were constrained with the SHAKE algorithm (tolerance 10^–8^ Å), and the equations of motion were integrated using the velocity-Verlet scheme with a time step of 0.5  fs. Each trajectory consisted of a 0.5 ns equilibration phase, followed by a 0.5 ns production run. Although this production length is shorter than typically required for converged thermodynamic or transport property evaluations, here the MD serves solely to generate diverse, thermally equilibrated snapshots for subsequent DFT calculations. For this purpose, extensive long-time sampling is not needed; nevertheless, we verified that structural and energetic quantities (e.g., temperature and potential energy) were stable over the production window. Snapshots were extracted at timesteps 0, 500,000, and 1,000,000 from the production run, giving six configurations (two configurations from each frame). All simulations were conducted in the *NVT* ensemble using a Nosé–Hoover thermostat chain with three thermostats and a damping parameter of 50 fs.^58^ Periodic boundary conditions were applied in all three directions. All input scripts, potential files, output logs, convergence plots, and extracted snapshot coordinates are provided in the Supporting Material, including full details of: LAMMPS version/build settings, random seeds, boundary conditions, neighbor list settings, thermostat parameters, and force-field coefficients, enabling complete reproduction of the MD trajectories.

To validate the applicability of the obtained IFFs on large scales, MD simulations were performed as described by Li et al.^59^ A three-layer Pt(111) (Pt_3_Ni­(111) and Pt_3_Co­(111) slabs for alloys) slab with 10 × 10 surface cells was constructed, with 16 O_2_ molecules adsorbed randomly and 600 H_2_O molecules above. A 40 Å vacuum was included above the water slab. The system was energy minimized, equilibrated for 200 ps at 300 K, and run for 2.8 ns in the NVT ensemble using a Nosé–Hoover thermostat (τ = 0.2 ps), a 1 fs time step, and velocity-Verlet integration; snapshots were saved every 1ps.

### Fitting Procedure and Selection Criteria

The fitting procedure is illustrated in Figure 3, adapted from Valadez Huerta and Raabe.^16^ To avoid confusion with the term “iterations,” which typically refers to optimization algorithm steps, we use “cycle” to describe the parametrization procedure. This process involves iterative data set enrichment aimed at minimizing the data set size required for optimization. In the first cycle, configurations are generated using mixing rules applied to literature FFs, as described above. These configurations, together with the resulting interatomic forces and adsorption energies, form the first training data set for benchmarking.

**Figure 3:**
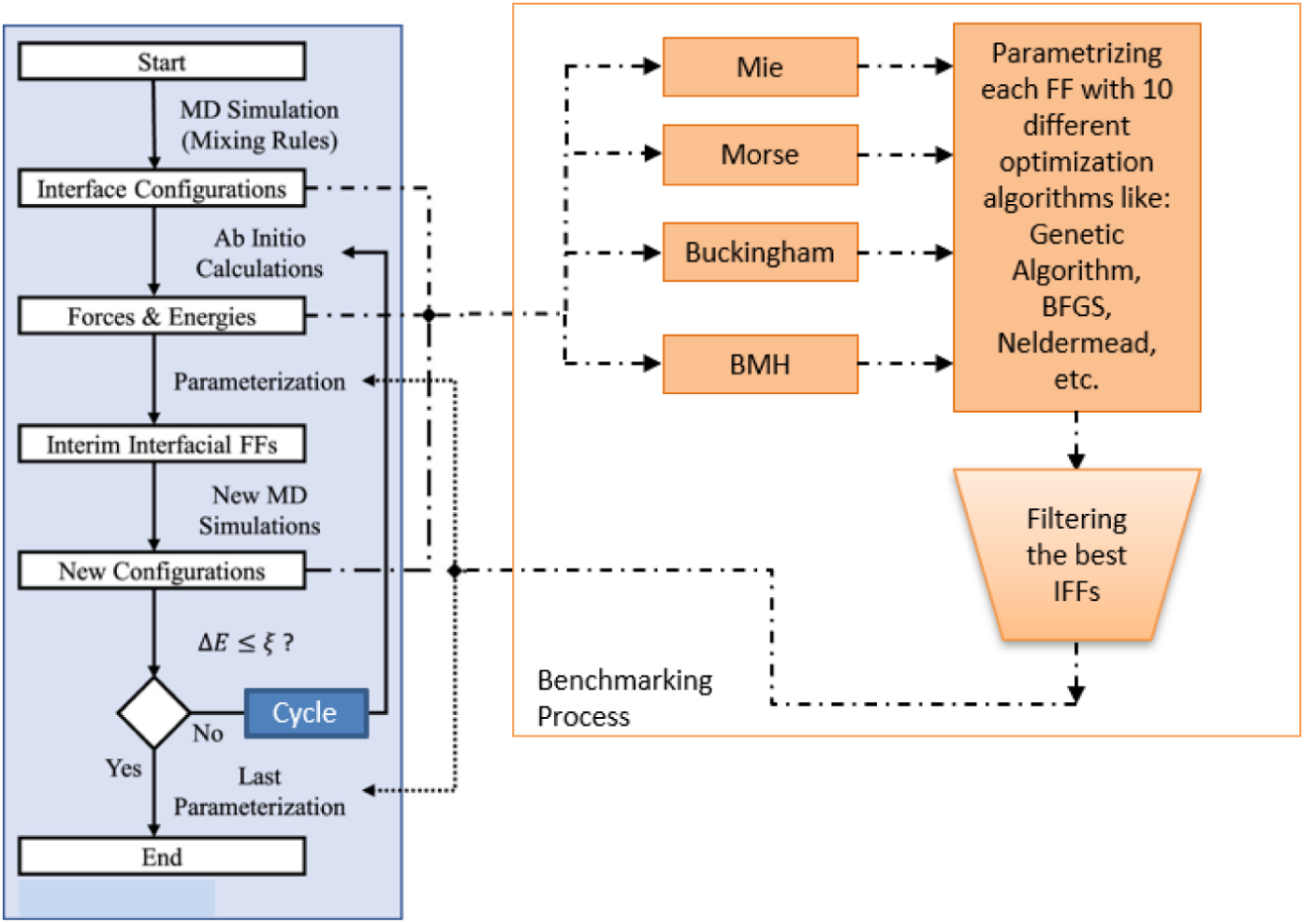
Procedure for fitting IFFs. A “cycle” (solid arrows within the blue block) refers to a parametrization and data set enrichment process. The benchmarking process (orange block) evaluates different force field forms and optimization algorithms. Dotted and dashed-dotted arrows indicate the interfaces between cycles and the benchmarking process.

During parametrization, the objective function *Z* (eq 10) is minimized at each optimization step. Here, *E* is the adsorption energy for a configuration, and *f_d_*,_*i*_ is the adsorption force component (in *d* ∈ {*x*, *y*, *z*}) for fluid atom *i* at the interface. All values are normalized to ab initio references (superscript 0) and weighted by *w*_*i*_, with adsorption energies weighted twice compared to force terms. Adsorption energies and forces are calculated as the difference between the adsorbed system and the sum of the isolated slab and fluid, defined formally in eqs 11 and 12.

10 $$Z=w_{e}{\left(\frac{E-E^{0}}{E^{0}}\right)}^{2}+\overset{N_{M}}{\underset{i=1}{\sum }}\underset{d\in \left\{x,y,z\right\}}{\sum }w_{f_{d}}{\left(\frac{f_{d,i}-f_{d,i}^{0}}{f_{d,i}^{0}}\right)}^{2}$$

11 $$E^{0}=E_{\mathrm{slab}+\mathrm{fluid},\mathrm{adsorbed}}-\left(E_{\mathrm{slab},\mathrm{isolated}}+E_{\mathrm{fluid},\mathrm{isolated}}\right)$$

12 $$f_{d,i}^{0}=f_{d,i}^{\mathrm{slab}+\mathrm{fluid},\mathrm{adsorbed}}-\left(f_{d,i}^{\mathrm{slab},\mathrm{isolated}}+f_{d,i}^{\mathrm{fluid},\mathrm{isolated}}\right)$$

Ten optimization algorithms were employed to parametrize each FF in the first cycle. These include both local and global strategies, selected for their ability to handle bounds, nonlinear constraints, and derivatives. All local optimization methods use multiple random initializations to reduce the risk of convergence to local minima. For implementation, the scipy.optimize.minimize^60^ module handles the first nine algorithms; the genetic algorithm (GA) is adapted from Valadez Huerta and Raabe.^16^ Table 3 summarizes key characteristics of the optimization algorithms.

**Table 3:** Summary of Optimization Algorithms Used for Force Field Parameterization^a^ ^b^

**Algorithm**	Supports Bounds	Nonlinear Constraints	Uses Gradient	Type
BFGS^61^	No	No	Yes	Local
CG^61^	No	No	Yes	Local
TNC^62^	Yes	No	Yes	Local
Powell^63^	Yes	No	No	Local
Nelder–Mead (NL)^64^	No	No	No	Local
Differential Evolution (DE)^65^	Yes	No	No	Global^c^
SHGO^66^	Yes	Yes	Partial^d^	Global^e^
DIRECT^67^	Yes	No	No	Global^f^
Dual Annealing (DA)^68^	Yes	Partial	Partial	Global
Genetic Algorithm (GA)^16^	Yes	Yes	No	Global^g^

a All local methods are executed with multiple starts.

b “Partial” indicates optional or limited support by implementation.

c Evolutionary algorithm inspired by biological evolution.

d “Partial” indicates optional or limited support depending on implementation.

e Deterministic.

f Stochastic.

g See text.

A multistart strategy is applied to all local optimization algorithms. Four strategies are used for initial guess generation: (i) the midpoint of parameter ranges; (ii) half of the parameters at lower and half at upper bounds; (iii) the inverse of (ii); (iv) random values within the allowed ranges.

Sen et al. reported a significant improvement in accuracy using 200 initial guesses for optimizing nine parameters. Increasing the number beyond 200 (to 300, 400, or 500) did not yield additional benefits, corresponding to about 22 initial guesses per parameter.^28^ Accordingly, we used 1000 initial guesses to accommodate FFs with more parameters and more interactions.

The choice of the stopping criterion is crucial, as optimization algorithms may measure progress by function evaluations, iterations, or CPU time. For benchmarking, we use iterations: optimization terminates when either (i) the change in the objective function between iterations drops below *τ* = 10^–12^, or (ii) the number of iterations reaches 10,000 ${(N}_{\mathrm{Iterations}}^{\max}=10,000)$ . Many algorithms involve additional hyperparameters (e.g., Nelder–Mead’s xatol/fatol as implemented in SciPy^60^), which are set to SciPy defaults (see in Table  S2). All GA hyperparameters (population size, crossover weighting, meta-mutation, etc.) follow Valadez Huerta and Raabe.^16^

Each individual IFF is defined by a unique combination of an optimization algorithm and an FF model; e.g., Born–Mayer–Huntins FF optimized with BFGS is BFGS_BMH. With 10 optimization algorithms and 4 different FFs, 40 distinct IFFs were considered in the first cycle. MD simulations with each IFF generate new configurations for which adsorption energies and forces are computed via DFT (data set enrichment).^16^ Each IFF’s quality is assessed by the mean absolute error (MAE) vs ab initio adsorption energies and forces:

13 $$\mathrm{MAE}=\frac{1}{N}\overset{N}{\underset{i=1}{\sum }}\left|y_{i}^{\mathrm{pred}}-y_{i}^{\mathrm{ref}}\right|$$

where *y* is the property (adsorption energy or forces). It should be emphasized that, unless stated otherwise, the MAEs for energies reported in this work are calculated *per configuration*, i.e., for the total adsorption energy of all fluid molecules present in that configuration and not per individual molecule. Because DFT calculations are expensive, not all 40 IFFs are enriched, but we select only IFFs with MAE below 3 eV in the first cycle. Additionally, we include two IFFs with higher MAE (5–6 eV) in the following cycles to test enrichment effects. Enrichment is repeated until the change in adsorption energy between cycles is below 1 × 10^–4^ eV; the procedure then terminates.

#### DeepMD Model Training

To evaluate the feasibility of machine-learning-based interface force fields within our data regime, we trained three distinct DeepMD models using the DeepMD-kit package.^38^ The training data set comprised all configurations from all data set enrichment cycles over all different IFFs for the Pt–H_2_O interface. In total, 252 configurations (sum over six cycles for each of the seven selected initial IFFs) were used for model training. These configurations include both atomic coordinates and reference DFT energies and forces. Three model variants were trained:

DeepMD-TEF: trained from scratch on total energies and atomic forces for all atoms in each configuration.
DeepMD-AE: trained from scratch on adsorption energies and adsorption forces.
DeepMD-FT: fine-tuning of a pretrained model (DPA-3.1–3M)^69^ on our total energies and forces.

Each DeepMD model was trained using the standard descriptor–fitting network architecture, with the fitting network composed of three hidden layers containing 120 neurons each and using the tanh activation function, and the descriptor network composed of three hidden layers with 25 neurons each. The local environment was described using a cutoff radius of 8.0 Å, with smoothing beginning at 5.5 Å. The loss function was weighted as *wE* = 2.0 for energies, *wF* = 1.0 for forces, and *wV = 0* for the virial term, which was not used. The Adam optimizer was employed, starting with a learning rate of 1 × 10^–3^ and decaying exponentially to 1 × 10^–8^ over a total of 2,000,000 training steps. Training was performed with a batch size of two configurations, and all models were run for 2,000,000 steps. We note that our data set comprises 252 configurations, which is small by ML-FF standards. The network architecture employed here corresponds to the default DeepMD fitting/descriptor sizes chosen for comparability with prior DeepMD-based potentials. While this size offers sufficient capacity to represent complex interfacial environments, we mitigate overfitting risks by employing data augmentation, shuffling at each epoch, using a held-out validation set, and, for fine-tuned models, leveraging pretrained weights from DPA-3.1–3M.^69^ Smaller architectures were also tested, yielding similar qualitative trends in MAE but no systematic improvement in test-set accuracy; hence, the default architecture was retained.

Input configurations were randomly shuffled at each epoch, and 10% of the data set was held out for validation. Symmetry-preserving data augmentation was applied via random rotation around the surface normal and small translations to avoid overfitting to specific adsorption geometries. The resulting models were evaluated against an independent test set, the MAEs for which are given in Table 4, allowing comparison to classical IFF performance under identical data constraints.

**Table 4:** Comparison of MAE Values for Adsorption Energy and Force for Different Interface Force Fields (IFFs) from the Literature, This Work, as well as DeepMD^a^ ^b^

**FF Model**	**MAE Energy (eV)**	**MAE Forces (eV/Å** ^**2**^ **)**
BFGS_BMH (this work)	0.358	0.131
NL_BMH (this work)	0.356	0.142
GA_MO (this work)	0.359	0.133
DeepMD-TEF	22.07	0.563
DeepMD-AE	1.717	0.636
DeepMD-FT	11.226	0.475
COMB3 ^70^	1.20	0.253
LJ-Mixing rules (METAL^9^ + F3C^40^)	2.45	0.243
ReaxFF^71^	3.53	0.234
GAL17 ^17^	0.115^c^	–

a MAE values for IFFs developed in this work are calculated on the *test set* of configurations, whereas values for literature force fields are from single-set evaluations.

b For DeepMD, results are shown for three variants: DeepMD-TEF (trained from scratch on total energies and forces), DeepMD-AE (trained from scratch on adsorption energies and forces), and DeepMD-FT (fine-tuned from a pretrained model)—all evaluated on the same test set used for classical IFF comparisons.

c MAE value taken from literature.^17^

## Results and Discussion

### Benchmarking Optimization Algorithm and Force Field Combinations

Initial benchmarking of the optimization algorithms and force fields (FFs) was performed for the Pt–H_2_O interface. The benchmark results are presented first, followed by validation of the selection strategy by generating IFFs for additional alloy surfaces as well as for H_2_O and O_2_ fluids. All of the final optimized parameters for the IFFs are given in Table S4.

Figure 4 shows the typical progression of the objective function *Z* during the parametrization of the Morse FF optimized with the Nelder–Mead (NL) algorithm using a multistart approach. In this example, 1000 initial guesses were used, each with a maximum of 10,000 iterations, for the Pt–H_2_O interface.

**Figure 4:**
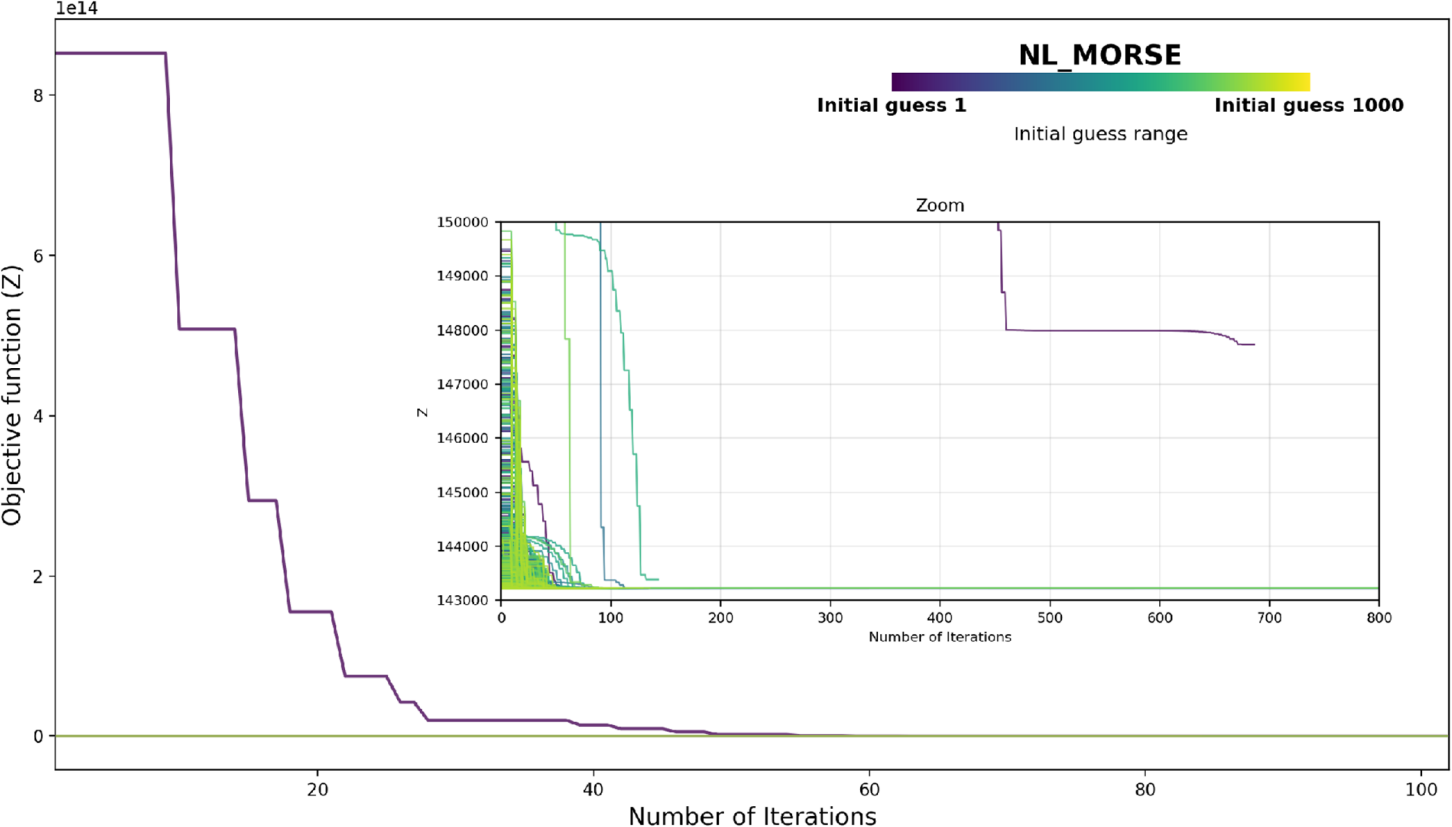
Progression of the objective function *Z* with respect to the number of iterations during the optimization of a Morse (MO) force field by using Nelder–Mead (NL) for the Pt–H_2_O interface. The main plot shows optimization trajectories from 1000 different initial parameter guesses; the inset zooms in on the initial region to illustrate the multistart scheme.

As depicted in Figure 4, the optimization run initiated from the midpoint of the parameter ranges (“Initial guess 1,” shown in dark purple) starts with an exceptionally high objective function value and converges much more slowly than the other initializations. Even after 700 iterations, this run fails to reach the lowest objective value obtained by the majority of other runs. In contrast, most other initial guesses (green/yellow lines) rapidly converge—typically within 50 iterations—to low objective function values clustered near 1.43 × 10^5^, as highlighted in the inset. While some initial guesses result in slower convergence or slightly higher plateaus, increasing the number of trials (1000) and allowing up to 10,000 iterations per run facilitate a thorough exploration of the parameter space. Because true global optimality cannot be guaranteed, we report the “best” or “lowest” objective value found among all runs.

As described previously, the first cycle of parametrization produces 40 distinct IFFs, each fitted using six configurations containing a total of 76 H_2_O molecules adsorbed on the slab surface. For each configuration, both the adsorption energy (1 data point) and all adsorption–force components (3*N* data points, where *N* is the number of fluid atoms in the adsorption region) were included in the fitting procedure for a given IFF in the first cycle. The resulting mean absolute error (MAE) values for adsorption energies and forces from this initial cycle are listed in Table S3 . Following the selection criteria defined earlier, the seven best-performing IFFs were retained for subsequent data set enrichment and reparameterization cycles until convergence was reached, as outlined above. Convergence occurred after six cycles for IFFs’: GA_MO, GA_BK, and NL_BMH, with each cycle comprising six configurations (36 total configurations across the training). The number of H_2_O molecules adsorbed varies between IFFs and cycles, ranging from 443 to 462—this set is referred to herein as the training set. The evolution of MAEs for these seven IFFs is shown in Figure 5, and the corresponding parity plots in Figure 6 are based on the training data. To evaluate transferability, an independent test set of six configurations containing a total of 81 adsorbed H_2_O molecules was generated. MAE values for adsorption energies and forces on this test set are reported in Table 4.

**Figure 5:**
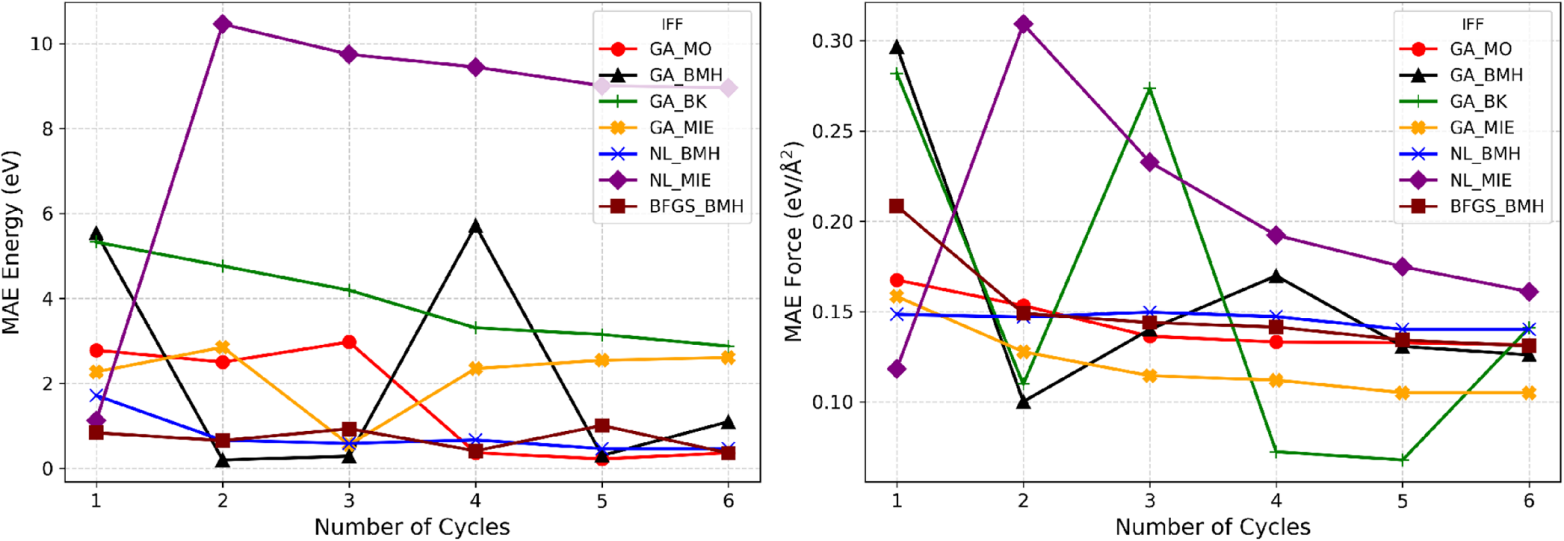
Evolution of the mean absolute error (MAE) in adsorption energies (left) and forces (right) for seven IFFs over six enrichment–reparameterization training cycles.

**Figure 6:**
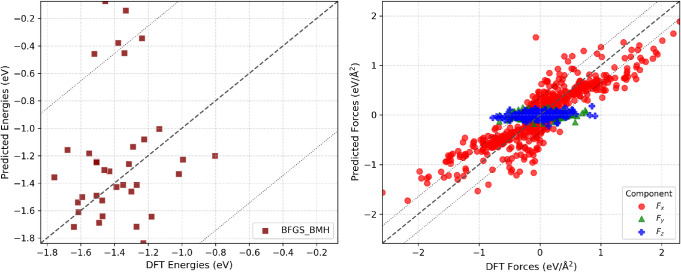
Comparison between DFT and predicted adsorption energies (left) and force components (right) for the BFGS_BMH IFF for the Pt–H_2_O interface over six cycles of the training set. The diagonal dashed line in both figures indicates perfect agreement, while the surrounding dashed lines mark the 95% confidence interval.

Figure 5 (left) shows that gradient-based optimization of BMH FFs (NL_BMH and BFGS_BMH) rapidly converges below 1 eV by the second cycle and remains stable, while NL_MIE never drops below 9 eV. The GA-based IFFs (GA_MO, GA_BMH, GA_MIE) all start at 2–3 eV and improve slowly; GA_BMH exhibits a spike in cycle 4 but none reach the accuracy of NL and BFGS-optimized BMH. Only GA_BK shows a monotonic decline from ∼5 eV to ∼3 eV over six cycles. In Figure 5 (right), GA_MIE yields the lowest force error (∼0.11 eV/Å^2^), while NL_MIE is the worst. GA_BK shows a significant dip at cycle 5 (∼0.07 eV/Å^2^), and the NL_BMH/GA_MO plateau near 0.14 eV/Å^2^. These results indicate that BMH force fields, with their larger number of adjustable parameters, can be accurately optimized for both energies and forces using gradient-based methods. The optimization of forces exhibits particularly smooth convergence because each configuration in the training set provides 3*N* force components (for *N* atoms), as opposed to just one energy value per configuration. This abundance of force data points enhances statistical averaging during training and makes the convergence of forces appreciably smoother than that of energies, as observed across different force field models.

The scatter plots in Figure 6 demonstrate that the BFGS_BMH IFF yields a close linear correlation between the predictions and DFT values for both energies and forces. Dotted lines indicate the 95% confidence interval, highlighting the spread around ideal agreement. For energy, predictions generally fall within a narrow range but with some scatter; for forces, *F*_*x*_ spans broadly and tracks DFT results, while *F*_*y*_ and *F*_*z*_ remain clustered near zero, reflecting their weaker variation—most likely owing to dissociative forces on adsorbed molecules at the reactive Pt surface, which are not captured by the tested FFs. BFGS_BMH captures the main trends but shows systematic deviations beyond the confidence bounds, especially for challenging force components.

The IFFs available from the literature, the BFGS_BMH, NL_BMH, and GA_MO models developed here, and the three DeepMD variants were used to predict adsorption energies and forces for all 252 configurations generated over six enrichment cycles from seven IFFs. The GAL17 model could not be tested directly due to the lack of a suitable LAMMPS implementation.^17^ Table 4 summarizes the resulting MAEs. Our BFGS_BMH IFF delivers the lowest error except for GAL17,^17^ for which a lower energy MAE is reported in the original work by Steinmann et al. The high accuracy of GAL17 is reasonable: it consists of 12 parameters, covers multiple rotational water states, and was trained on a much larger set (∼210 configurations). Our aim here is to propose a transferable, data-efficient protocol for parametrizing IFFs for broader application. The mixing-rules LJ model yields a high error and should be avoided for large-scale MD.

For the DeepMD models, although all were trained on the complete available data set, their substantial energy and force errors highlight the limitations of applying machine-learning potentials in our low-data regime. As emphasized in a recent review by Unke et al.,^7^ ML-based force fields generally require large and diverse data sets to achieve high accuracy; typical benchmarks, such as the MD17 molecular data set, show significant performance variation with training set size. For complex interfaces, the required data set size is even greater. For example, Chang et al.^72^ used DP-GEN to develop a DeepMD potential for the AlLi–AP (ammonium perchlorate) interface—featuring a very small adsorbate—requiring 11,251 configurations for satisfactory accuracy and compiled similar data set sizes from prior ML-based interface studies. Scaling such an approach to the large, flexible adsorbates and multicomponent interfaces would be computationally impractical. Accordingly, the DeepMD results here should be interpreted as a compact, exploratory comparison rather than a full-scale ML model development. We reiterate that ML-based IFFs are promising for interfacial systems when sufficient, diverse training data are available, but our contribution in this study is complementary—offering a physically grounded, data-lean classical IFF approach that achieves competitive accuracy under tight data constraints.

### DFT Validation of IFFs for Pt–H_2_O Interface

Based on the above error metrics, we selected the GA_MO, BFGS_BMH, and NL_BMH force fields for direct comparison with DFT, also including results from the mixing-rules force field for reference. To assess the IFFs’ ability to reproduce the most stable adsorbate geometries, we constructed three Pt(111)–vacuum model systems: single water monomer, dimer, and trimer, each placed at random heights above the slab and relaxed with each IFF.

Figure 7 shows that both NL_BMH and BFGS_BMH produce well-defined, stable adsorption for all species; MixingRules and GA_MO do not discover the distinct high occurrence of correct adsorption geometry. In BFGS_BMH and NL_BMH, water molecules adopt near-flat orientations (0–30^°^ tilt) matching DFT predictions (monomer: 0–8°; dimer: ∼16°; trimer: ∼20^°^).^73^ Pt–O distances for these geometries are 2.8–3.5 Å, close to DFT values of 2.34 Å (monomer), 2.2–2.26 Å (dimer), and 2.18–3.60 Å (trimer). Such validation is vital: Fu et al. have shown that even low-force-error models can yield nonphysical structures and unreliable trajectories over long time scales.^34^

**Figure 7:**
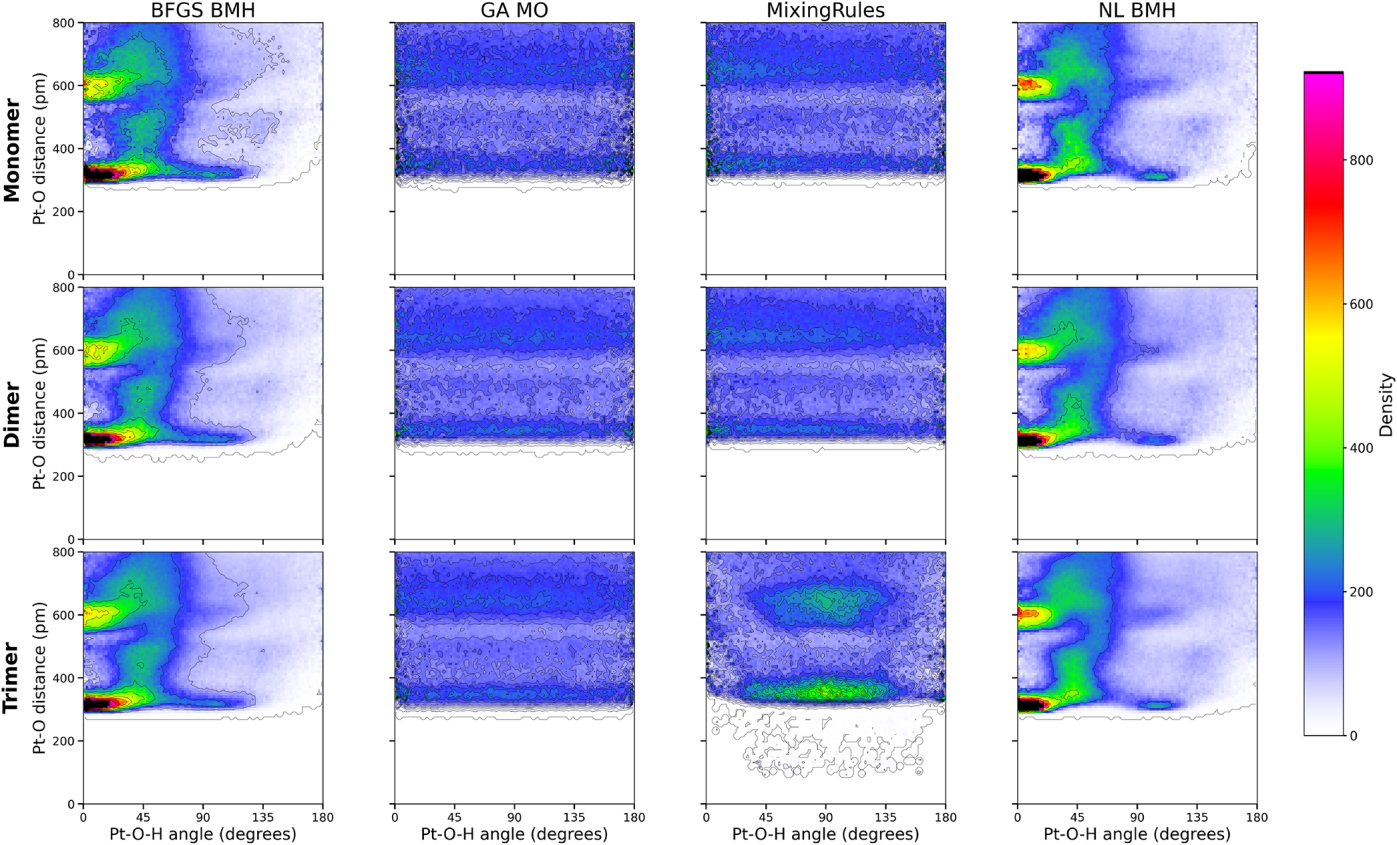
Two-dimensional density maps of the distance between Pt and O versus Pt–O–H angles (between the O–H bond and the slab normal) for water monomer, dimer, and trimer on Pt(111), as predicted by BFGS_BMH, GA_MO, NL_BMH, and mixing rules IFFs. Contours show equiprobability; color indicates normalized density (arb. units).

### IFF for Pt–O_2_ Interface

Following the same workflow and selection criteria, the BMH force field, parametrized using the Nelder–Mead algorithm (NL_BMH) was identified as the best-performing IFF for the Pt–O_2_ interface. Convergence was achieved after 11 reparameterization cycles, each comprising six configurations, for a total of 66 configurations containing 528 adsorbed O_2_ molecules. Figure 8 presents comparisons between the adsorption energies and forces predicted by this model and those obtained from the DFT calculations for all training configurations.

**Figure 8:**
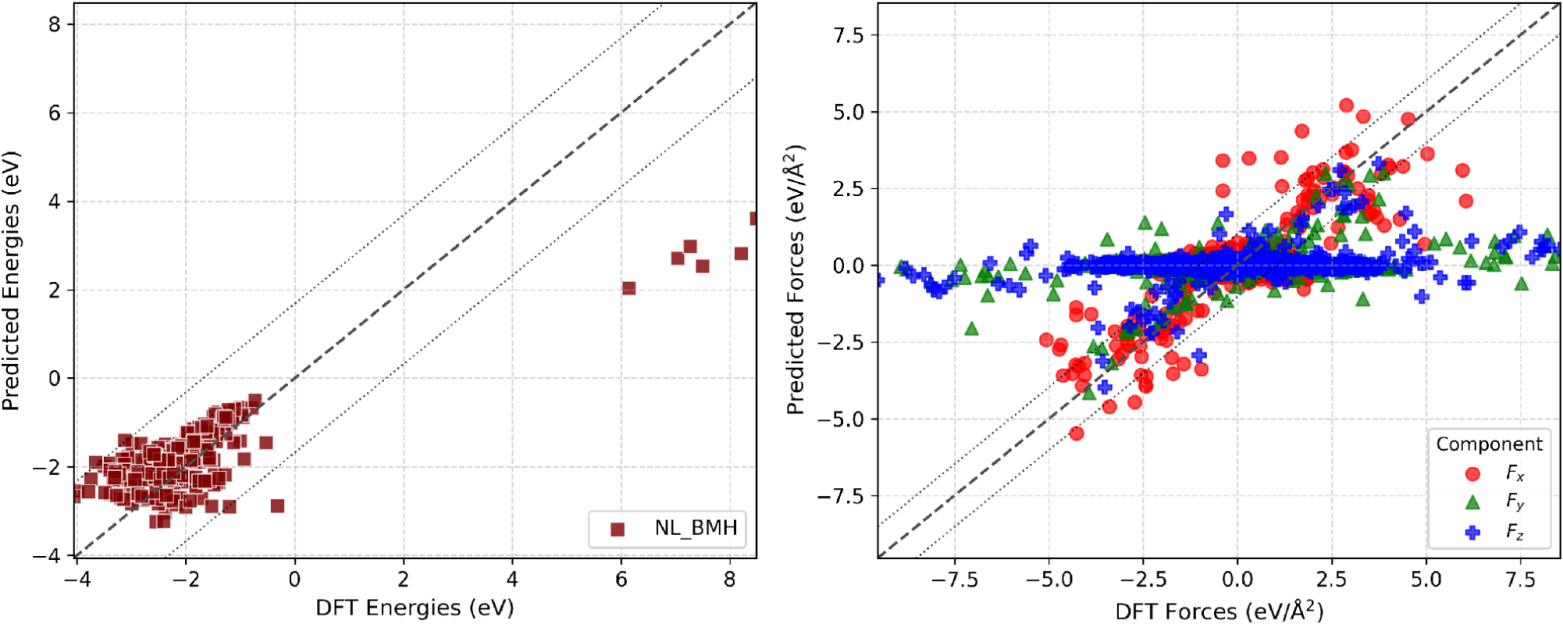
Comparison of adsorption energies (left) and force components (right) predicted by the NL_BMH IFF for the Pt–O_2_ interface with DFT reference values for all training configurations. The diagonal dashed line denotes perfect agreement; outer dashed lines show the 95% confidence interval.

The IFF predicts adsorption energies accurately, except for certain outlier configurations where O_2_ is unphysically close to Pt, an issue systematically corrected during reparameterization. As seen in Figure 8, surface-normal forces (*F*_*x*_) are well reproduced, but lateral forces (*F*_*y*_, *F*_*z*_) show low DFT correlation. This anisotropy reflects the strongly directional energy landscape for O_2_ on Pt(111), as explained by Carbogno et al.,^74^ where attractive and repulsive normal interactions vary quickly, but lateral interactions remain flat or ambiguous. For static DFT calculations, small lateral forces are susceptible to numerical noise from finite convergence thresholds, introducing an artifact in the adsorption force analysis (cf. eq 12). Table 5 summarizes the MAEs for all interfaces. Compared to mixing-rules-based IFFs, the present IFF shows much smaller errors in energies and forces (Table 5); for clarity, mixing-rules predictions are omitted from the scatter plots.

**Table 5:** Mean Absolute Errors (MAEs) for Adsorption Energies and Forces Calculated for the Configurations in the Test Set Using IFFs Developed in This Work and LJ-Mixing-Rules Force Fields for Selected Pt Alloy Interfaces^a^

			IFF		LJ-Mixing rules	
**Interface (IFF)**	Train Configs	Test Configs	Energy (eV)	Force (eV/Å^2^)	Energy (eV)	Force (eV/Å^2^)
Pt–H_2_O (BFGS_BMH)	36	6	0.32	0.12	1.93	0.16
Pt_3_Ni–H_2_O (NL_BMH)	75	6	0.83	0.18	47.44	1.89
Pt_3_Co–H_2_O (NL_BMH)	24	6	1.05	0.14	4.43 × 10^6^	2.76 × 10^5^
Pt–O_2_ (NL_BMH)	66	6	0.41	0.85	2.92	1.44
Pt_3_Ni–O_2_ (BFGS_BMH)	84	6	0.30	0.42	2.16	0.99
Pt_3_Co–O_2_ (BFGS_BMH)	15	6	0.68	0.67	0.85	0.75

a The number of configurations in the training and test sets is also listed for each IFF from this work.

To validate the applicability of the BFGS_BMH force field for Pt–H_2_O and NL_BMH for Pt–O_2_ on large scales, MD simulations were performed as by Li et al.^59^ Parallel and nonparallel configuration definitions follow Li et al.^59^

Figure 9 (left) shows that, consistent with experiment and DFT studies^75,76^ the IFFs predict O_2_ molecules to reside significantly closer to the Pt(111) surface than H_2_O, in contrast to the nearly equidistant layering observed when using simple LJ-mixing rules^59^ (see Table 6). Furthermore, the BFGS_BMH and NL_BMH force fields produce a much greater fraction of parallel configurations for both H_2_O and O_2_ at the interface, yielding more realistic molecular orientations and greater stability of the adsorbed species. These improvements underscore the superior accuracy and physical fidelity achieved by the present IFF parametrization approach when compared to employing simple mixing rules.

**Figure 9:**
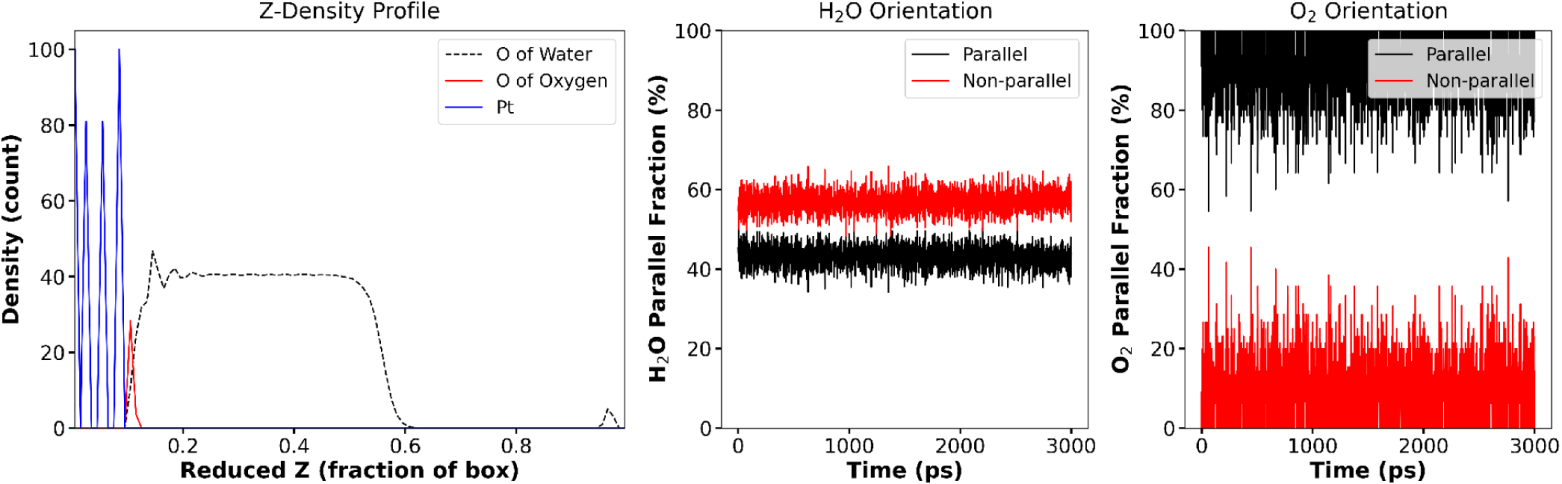
(Left) *Z*-density profile for Pt (blue), O in O_2_ (red), and O in H_2_O (dashed black); (middle) distribution of parallel (black) and nonparallel (red) H_2_O configurations; (right) similar orientation distribution for adsorbed O_2_.

**Table 6:** Comparison of the O_2_ and H_2_O Adsorption Configurations on Alloy Slabs and Pure Pt Surface for Both Optimized IFF and LJ-Mixing-Rule-Based IFFs^a^

	O_2_ % orient. parallel		H_2_O % orient.parallel		O_2_–surface distance (Å)		H_2_O–surface distance (Å)	
Slab	IFF	LJ-mix	IFF	LJ-mix	IFF	LJ-mix	IFF	LJ-mix
Pt	89.20	70.00^b^	42.88	55.00^b^	2.68	3.66^b^	3.43	3.66^b^
Pt_3_Ni	78.19	66.79	70.00	56.45	2.15	3.05	2.33	2.93
Pt_3_Co	83.94	66.91	75.36	0.00	2.40	3.11	2.87	2.99

a O_2_ and H_2_O orientation columns report the running average percentage of parallel configurations; distance columns refer to the average vertical distance (Å) from the first atomic layer of the slab for the respective adsorbate.

b Approximated values from ref. 59.

### Application for Alloys

Interface force fields (IFFs) were developed for bulk O_2_ and H_2_O adsorbed individually on alloy surfaces Pt_3_Ni and Pt_3_Co to assess the robustness of the parameterization strategy for complex surfaces. For the Pt_3_Co slab, the (111) surface was oriented normal to the *xy*-plane to address the anisotropies in the force prediction observed for the O_2_ adsorption. Figure 10 shows parity plots of adsorption energies and forces versus DFT calculations for all configurations in the training set. The mean absolute errors (MAEs) obtained for the separate test set are reported in Table 5. The training set size varies by interface, whereas the test set contains six configurations for each case. The NL_BMH and BFGS_BMH IFFs closely reproduce DFT adsorption energies across all alloy–molecule interfaces, as indicated by clustering along the parity line and consistently low MAEs. Adsorption forces for H_2_O show excellent DFT correlation, while larger deviations for O_2_ occur mainly in lateral components (*F*_*y*_, *F*_*z*_ for Pt_3_Ni and Pt_3_Co), with normal components remaining in strong agreement—mirroring the trends found for pure Pt. These results validate our IFF selection protocol and demonstrate its significant improvement over conventional mixing rules, which exhibit much greater errors (Table 5).

**Figure 10:**
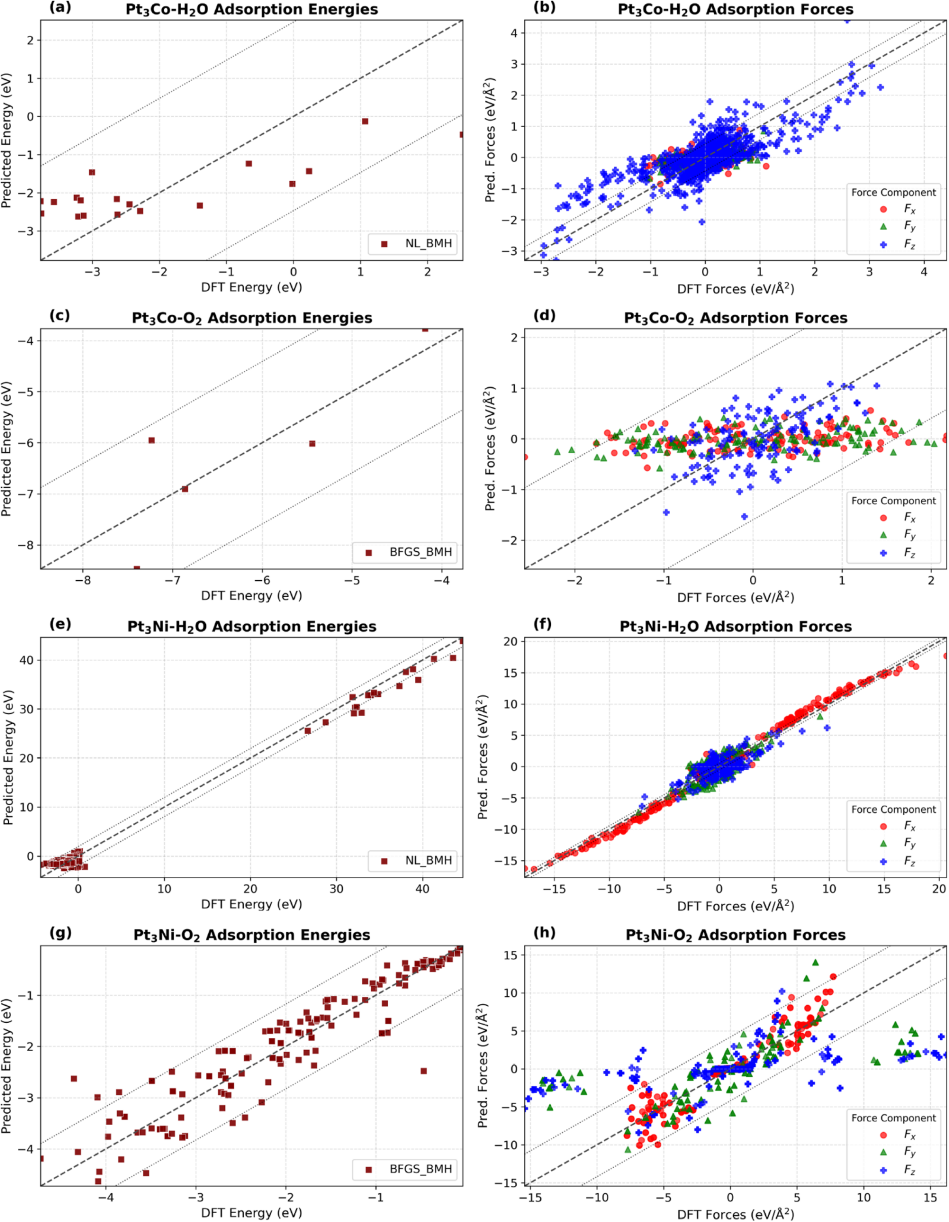
Parity plots comparing DFT and IFF-predicted adsorption energies (left) and force components (right) for four Pt alloy–molecule interfaces for the configurations in the training set: (a,b) Pt_3_Co–H_2_O (NL_BMH), (c,d) Pt_3_Co–O_2_ (BFGS_BMH), (e,f) Pt_3_Ni–H_2_O, (NL_BMH), and (g,h) Pt_3_Ni–O_2_ (BFGS_BMH). The dashed diagonal line indicates perfect agreement; dotted lines enclose the 95% confidence interval.

We evaluated the performance of the optimized IFFs for the different alloys by conducting MD simulations, following the approach of Li et al.^59^ The investigations were performed independently for Pt_3_Ni and Pt_3_Co slabs. The orientation and adsorption characteristics of O_2_ and H_2_O on pure Pt, Pt_3_Ni, and Pt_3_Co slabs are summarized in Table 6. We report the running average percentage of parallel configurations and the average vertical distance of adsorbates from the first atomic layer of each slab.

The results in Table 6 demonstrate clear differences between the optimized IFFs and the LJ-mixing-rules IFFs, as well as systematic trends across pure Pt and alloy surfaces. First, comparing the two force field parametrizations, the optimized IFFs predict a substantially higher fraction of parallel adsorption configurations for both O_2_ and H_2_O across all surfaces, particularly for H_2_O on Pt_3_Co, than do the LJ-mixing-rules IFFs. Additionally, the optimized IFFs predict shorter vertical adsorption distances for both adsorbates, with O_2_ consistently adsorbing closer to the metal surface than H_2_O—a trend less pronounced when using the LJ-mixing rules. These improvements indicate that the optimized IFFs yield adsorption geometries more consistent with expected surface reactivity.^77^ Second, when considering the effect of alloying, both force field approaches predict that alloyed surfaces (Pt_3_Ni and Pt_3_Co) facilitate closer adsorption of O_2_ and H_2_O compared with pure Pt. Notably, while pure Pt exhibits the largest fraction of parallel O_2_ configurations, the alloys show a higher fraction of parallel H_2_O configurations in comparison to pure Pt. This shift highlights a notable change in adsorption orientation on alloyed versus pure Pt surfaces. Overall, the trends predicted with the optimized IFFs—specifically the closer and more parallel adsorption of molecules—are in agreement with previous DFT studies for Pt_3_Ni,^77^ supporting a more realistic description of molecular adsorption relevant to catalytic activity.

## Conclusion

In this work, we have systematically benchmarked a suite of classical force field functional forms and optimization algorithms for the parametrization of interface force fields (IFFs) in metal–fluid systems, using Pt interfaces with water and molecular oxygen as representative cases, and then applied it to Pt_3_Ni, and Pt_3_Co. Our protocol, combining multistart local and global optimization schemes, enables reliable identification of optimal force field parameters with rigorous MAE control and minimal DFT training data. The resulting IFFs, particularly those based on the BMH form and optimized with gradient-based methods, consistently outperformed traditional mixing rules and several literature FFs, exhibiting excellent agreement with DFT reference energies and forces across both pure and alloy Pt surfaces. Rigorous DFT and MD validation demonstrates that these models accurately capture key structural motifs and interfacial energetics, offering robust predictions even in complex alloy environments. Force matching with DFT-based data set enrichment was shown to be critical for achieving transferability. Comparison with neural network potentials underlines the competitive accuracy of classical IFFs when guided by a systematic fitting and selection workflow. These findings highlight the value of a benchmarking-driven approach for IFF development and provide practical guidelines for parametrizing transferable, high-fidelity IFFs for catalytic interfaces and related interfacial systems.

## Supplementary Material





## Notes

Complete Quantum ESPRESSO (QE) input files for DFT calculations, LAMMPS input scripts for classical MD simulations, and a custom Python script used for parametrization of interface force fields. The files are organized into separate folders by type, with a necessary README file describing the contents, directory structure, and step-by-step usage instructions (ZIP)
(a) Initial LJ parameters, (b) MAE results for 40 IFFs in the first cycle parametrization process for Pt–H_2_O interface, (c) final IFF parameters for all six interfaces (PDF)
