# Supplementary figures and images for: Developing Interface Force Fields for Water and Oxygen on Pt, and Pt 3 Ni, Pt 3 Co Alloy Surfaces for Proton-Exchange Membrane Fuel Cell (PEMFC) Applications
Source: ACS Omega. 2026 Feb 6;11(6):10649–63. doi: 10.1021/acsomega.5c11427 (PMC12917792; doi:10.1021/acsomega.5c11427)

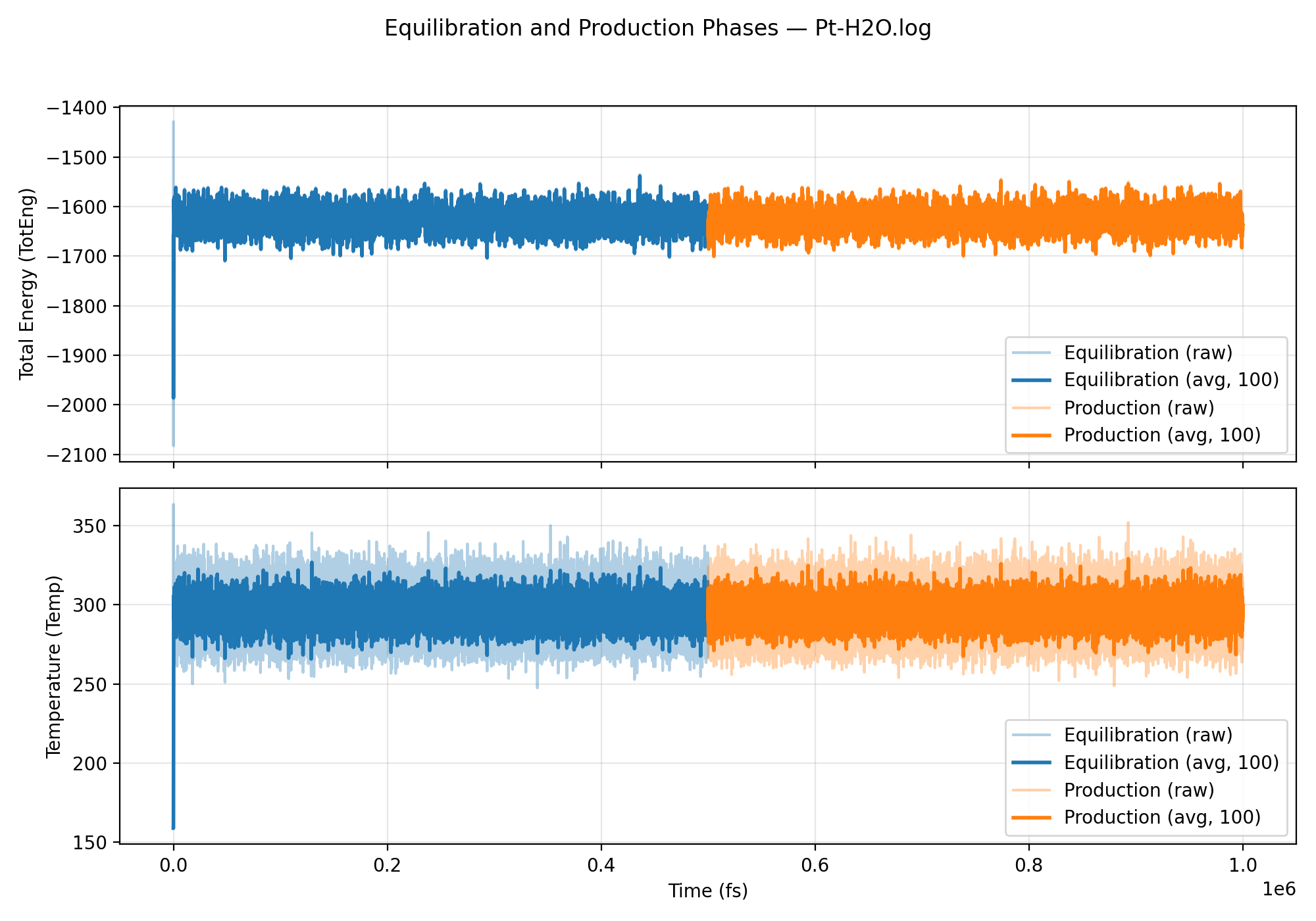

Supplement: Supplementary file 1 [file ao5c11427_si_001.zip › Supporting_material/01.Cycle01_config_generation_and_DFT_calculations/00.Log_plot.png]
