# Supplementary material for: Developing Interface Force Fields for Water and Oxygen on Pt, and Pt 3 Ni, Pt 3 Co Alloy Surfaces for Proton-Exchange Membrane Fuel Cell (PEMFC) Applications
Source: ACS Omega. 2026 Feb 6;11(6):10649–63. doi: 10.1021/acsomega.5c11427 (PMC12917792; doi:10.1021/acsomega.5c11427)
Supplement: Supplementary file 2 [file ao5c11427_si_002.pdf]

Supporting Information
Developing Interface Force Fields for Water and Oxygen on Pt, and Pt_3Ni, Pt_3Co alloy surfaces for Proton Exchange Membrane Fuel Cell (PEMFC) applications

Aditya S. Kale[†,‡] and Gabriele Raabe[*,†,‡]

†*Institut für Thermodynamik, Technische Universität Braunschweig, Braunschweig*

‡*Cluster of Excellence SE2A-Sustainable and Energy-Efficient Aviation, TU Braunschweig, Braunschweig, Germany*

E-mail: G.Raabe@tu-braunschweig.de

Initial LJ Parameters

Table S1: Lennard-Jones (LJ) parameters for different atom types used in this work. For the first cycle, arithmetic mixing rules ($\epsilon_{ij} = \sqrt{\epsilon_i \epsilon_j}$ and $\sigma_{ij} = (\sigma_i + \sigma_j)/2$) are used to calculate the LJ parameters for interactions between solid and bulk fluid atoms. The partial charges q_i are also shown; literature references as in the main manuscript.

Element Pair	ϵ (kcal/mol)	σ (Å)	q_i (e)	Literature
Pt–Pt	7.80	2.845	0	8
Pt_{Pt_3Ni}–Pt_{Pt_3Ni}	7.80	2.845	−0.6667	8
Ni–Ni	5.65	2.552	2.0, 2.0	8
Pt_{Pt_3Co}–Pt_{Pt_3Co}	4.641	2.410	−0.39	49
Co–Co	3.487	2.180	0.546	49
O_{H_2O}–O_{H_2O}	0.1848	3.5532	−0.82	39
H_{H_2O}–H_{H_2O}	0.01	0.90	0.410	39
O_{O_2}–O_{O_2}	0.1047	3.297	0	40

Scipy Hyperparameters

Table S2: Default values for unique hyperparameters in key `SciPy` optimizers. "BFGS", "CG", "Powell", "Nelder-Mead", "TNC" refer to `minimize(method=...)`; "DE" = Differential Evolution; "DA" = Dual Annealing; "DIRECT" = DIRECT; "SHGO" = SHGO.

Hyperparameter	Default	Algorithms
return_all	True	NL, Powell
xatol	1×10^{-8}	NL
fatol	1×10^{-8}	NL
xtol	1×10^{-4} (Powell), -1 (TNC)	Powell, TNC
ftol	1×10^{-4} (Powell), -1 (TNC)	Powell, TNC
direc	Full rank	Powell
gtol	-1 (TNC), 1×10^{-5}	BFGS, CG, TNC
eps	1.49×10^{-8} (BFGS/CG), 1×10^{-4} (DIRECT)	BFGS, CG, DIRECT
stepmx	0	TNC
accuracy	0	TNC
minfev	0	TNC
rescale	-1	TNC
eta	-1	TNC
strategy	best1bin	DE
popsize	15	DE
tol	0.01	DE, SHGO
mutation	(0.5, 1)	DE
recombination	0.7	DE
seed	12345	DE, DA
polish	True	DE
updating	immediate	DE
workers	1	DE, SHGO
locally_biased	True	DIRECT

Hyperparameter	Default	Algorithms
f_min	$-\infty$	DIRECT
f_min_rtol	1×10^{-4}	DIRECT
vol_tol	1×10^{-16}	DIRECT
len_tol	1×10^{-6}	DIRECT
n	100	SHGO
iters	1	SHGO
sampling_method	simplicial	SHGO
f_tol (SHGO)	1×10^{-12}	SHGO
symmetry	False	SHGO
minimize_every_iter	True	SHGO
local_iter	False	SHGO
infty_constraints	True	SHGO
initial_temp	5230.0	DA
restart_temp_ratio	2×10^{-5}	DA
visit	2.62	DA
accept	-5.0	DA
no_local_search	False	DA

MAE for 1st Optimization Cycle of Pt-H_2O Interface

Table S3: Mean absolute error (MAE) for adsorption energy (E) and force (F) for each IFF optimized in the first cycle for Pt-H_2O interface.

FF	MAE E (eV)	MAE_F (eV/Å^2)
GA_MO	2.779798	0.167441667
GA_BMH	5.4532	0.296579333
GA_BK	5.326925	0.281878667
GA_MIE	2.269	0.158354
NL_BMH	1.714911	0.148427833
NL_MIE	1.12141	0.181180167
NL_BK	6.382047	0.50964
NL_MO	7.8944584	0.486488
BFGS_BMH	0.834247	0.208204667
BFGS_MIE	6.3254	0.486488
BFGS_BK	6.3254	0.453667
BFGS_MO	1.32265	1.658945
CG_BMH	8.8459	0.6823
CG_MIE	9.1284	0.7835
CG_BK	7.5437	0.5433
CG_MO	8.6543	1.1578
TNC_BMH	7.8012	0.6688
TNC_MIE	8.4411	0.7569
TNC_BK	9.1768	0.6095
TNC_MO	10.2345	1.2991
Powell_BMH	10.1102	0.8235
Powell_MIE	9.8721	0.6733
Powell_BK	9.4625	0.7642
Powell_MO	10.9816	1.3261
DE_BMH	11.1547	0.9281
DE_MIE	8.6781	0.6936
DE_BK	10.1237	0.7828
DE_MO	12.2456	1.0524
SHGO_BMH	10.8943	1.0162
SHGO_MIE	11.3425	1.1938
SHGO_BK	10.8529	0.9877
SHGO_MO	12.3658	1.5533
DIRECT_BMH	12.7411	0.9946
DIRECT_MIE	9.7653	0.8375
DIRECT_BK	10.5386	1.2744
DIRECT_MO	12.7789	1.6612
DA_BMH	13.3342	1.2638
DA_MIE	11.2397	1.0346
DA_BK	13.0172	1.5322
DA_MO	14.4615	2.2981
DeepMD	11.225513	3.860578111

Final IFF Parameters Optimized and Used for Validation

Table S4: Final optimized BMH potential parameters for respective interfaces.

Pair	A (kcal/mol)	ρ (Å^{-1})	σ (Å)	C (kcal/mol Å^6)	D (kcal/mol Å^8)
O_{H_2O}–Pt	28195.0601	0.2602	0.7484	699.9999	6.7709e-09
H_{H_2O}–Pt	0.0049	8.0429	0.7216	564.3035	699.9999
O_{O_2}–Pt	671010.4709	1.0889	1.1755	1737.2259	10000.0
O_{H_2O}–Ni_{Pt_3Ni}	6.7528e-07	0.1105	4.9988	667.702	697.416
O_{H_2O}–Pt_{Pt_3Ni}	0.00072	0.2765	5.0	700.0	71.0817
H_{H_2O}–Ni_{Pt_3Ni}	3.05946	0.318139	4.25021	626.531	19.2966
H_{H_2O}–Pt_{Pt_3Ni}	136.911	0.410383	0.9694	699.903	0.0946226
Pt_{Pt_3Ni}–O_{O_2}	2.57833×10^6	0.02609	1.68835	653.13049	264.93710
Ni_{Pt_3Ni}–O_{O_2}	2.94081×10^6	0.03820	2.82971	416.34386	385.79974
O_{H_2O}–Pt_{Pt_3Co}	1.06675e-05	0.151831	4.77526	700.00	0.00126656
O_{H_2O}–Co_{Pt_3Co}	1935.79	0.242937	2.69658	700	0.04754
H_{H_2O}–Pt_{Pt_3Co}	0.000933879	0.23659	3.77605	104.25	0.0167364
H_{H_2O}–Co_{Pt_3Co}	0.0247005	9.99606	0.81772	699.998	1.69282e-05
Pt_{Pt_3Co}–O_{O_2}	1.36170×10^6	0.02389	1.02057	1259.50593	1244.96919
Co_{Pt_3Co}–O_{O_2}	2.41907×10^6	0.08172	1.15085	1102.43789	5904.73776
